# Single-cell RNA sequencing reveals a peripheral landscape of immune cells in *Schistosomiasis japonica*

**DOI:** 10.1186/s13071-023-05975-y

**Published:** 2023-10-10

**Authors:** Junhui Li, Yu Zhang, Hao Li, Jie Jiang, Chen Guo, Zhaoqin Zhou, Yulin Luo, Chen Zhou, Yingzi Ming

**Affiliations:** 1 Transplantation Center, The Third Xiangya Hospital, Central South University, No. 138 Tongzipo Road, Changsha, 410013 Hunan China; 2 Engineering and Technology Research Center for Transplantation Medicine of National Health Commission, Changsha, Hunan China

**Keywords:** Single-cell RNA sequencing, *Schistosomiasis japonica*, Landscape of immune cells

## Abstract

**Background:**

Schistosomiasis, also known as bilharzia, is a devastating parasitic disease. This progressive and debilitating helminth disease is often associated with poverty and can lead to chronic poor health. Despite ongoing research, there is currently no effective vaccine for schistosomiasis, and praziquantel remains the only available treatment option. According to the progression of schistosomiasis, infections caused by schistosomes are classified into three distinct clinical phases: acute, chronic and advanced schistosomiasis. However, the underlying immune mechanism involved in the progression of schistosomiasis remains poorly understood.

**Methods:**

We employed single-cell RNA sequencing (scRNA-seq) to profile the immune landscape of *Schistosomiasis japonica* infection based on peripheral blood mononuclear cells (PBMCs) from a healthy control group (*n* = 4), chronic schistosomiasis group (*n* = 4) and advanced schistosomiasis group (*n* = 2).

**Results:**

Of 89,896 cells, 24 major cell clusters were ultimately included in our analysis. Neutrophils and NK/T cells accounted for the major proportion in the chronic group and the healthy group, and monocytes dominated in the advanced group. A preliminary study showed that NKT cells were increased in patients with schistosomiasis and that CXCR2 + NKT cells were proinflammatory cells. Plasma cells also accounted for a large proportion of B cells in the advanced group. MHC molecules in monocytes were notably lower in the advanced group than in the chronic group or the healthy control group. However, monocytes in the advanced group exhibited high expression of *FOLR3* and *CCR2.*

**Conclusions:**

Overall, this study enhances our understanding of the immune mechanisms involved in schistosomiasis. It provides a transcriptional atlas of peripheral immune cells that may contribute to elimination of the disease. This preliminary study suggests that the increased presence of CCR2 + monocyte and CXCR2 + NKT cells might participate in the progression of schistosomiasis.

**Graphical Abstract:**

**Figure:**
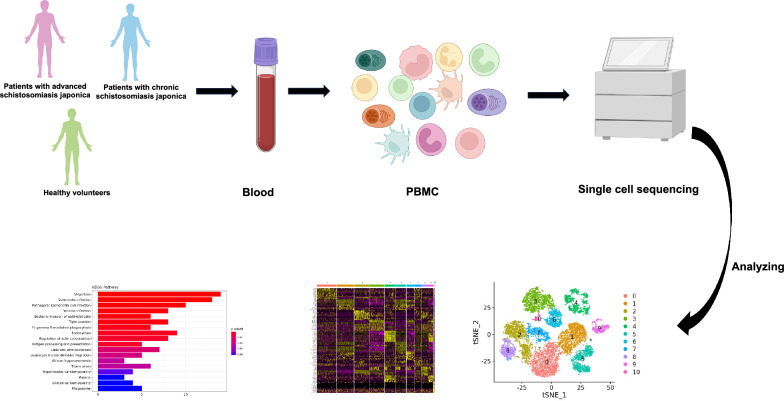

**Supplementary Information:**

The online version contains supplementary material available at 10.1186/s13071-023-05975-y.

## Background

Schistosomiasis is one of the most devastating parasitic diseases, affecting more than 250 million people worldwide [1]. Human schistosomiasis is mainly caused by *Schistosoma haematobium*, *S. mansoni* and *S. japonicum* [2]. *Schistosoma haematobium* and *S. mansoni* are predominantly present in Africa, the Middle East and South America, while schistosomiasis in China is primarily caused by *S. japonicum* [3]. This progressive and debilitating helminth disease is often linked to poverty and chronic poor health. Moreover, there is no effective vaccine, and the only available treatment is praziquantel, which cannot prevent reinfection and may lead to drug resistance. Therefore, elimination of schistosomiasis requires a deeper understanding of its pathogenesis and development of new therapeutic strategies against schistosome infection.

Schistosome infections are divided into three distinct clinical phases according to the progression of schistosomiasis: acute, chronic and advanced schistosomiasis [4]. Schistosome infection occurs in humans when in contact with fresh water contaminated by cercariae. Acute schistosomiasis, also known as Katayama fever, presents a range of symptoms, including fever, diarrhea, abdominal pain, fatigue and malaise [5, 6]. In chronic schistosomiasis, mature schistosomes produce many eggs, leading to immunopathological reactions and chronic inflammatory lesions [7]. *Schistosoma mansoni* and *S. japonicum* reside in the mesenteric veins and cause intestinal disease; *S. haematobium* resides in the pelvic venous plexus and is involved in lesions of the bladder wall [8, 9]. In advanced schistosomiasis, schistosome eggs, rather than adult worms, cause morbidity by driving formation of granulomas and fibrosis in the liver and intestinal tract [10]. Advanced schistosomiasis, which is associated with poor survival and prognosis, is usually accompanied by portal hypertension, ascites, splenomegaly and gastroesophageal variceal bleeding or granulomatous disease of the colon [11]. Although acute schistosomiasis is rare, chronic or advanced schistosomiasis is common in endemic areas. Unfortunately, there is currently no effective drug capable of preventing the transition from chronic schistosomiasis to advanced schistosomiasis.

Immunopathology plays a critical role in the development of schistosomiasis. The interaction between schistosomes and human immune cells is complex and is not fully understood. Both clinical and preclinical studies have shown that eosinophilia and increased IgE levels are hallmarks of the acute stage of the disease [12]. There is an obvious type 1 T helper cell (Th1) response to schistosome antigens, as characterized by increased levels of proinflammatory cytokines such as interferon gamma (IFN-γ), interleukin-1 (IL-1), tumor necrosis factor alpha (TNF-α) and IL-6. Following the production of eggs, soluble egg antigens (SEAs) stimulate a shift from Th1 to Th2 cell-dominant immunity, which is characterized by high levels of IL-4 and IL-10 [13, 14]. Th2-type cytokines such as IL-4, IL-5, and IL-13 are increased, whereas IFN-γ production is decreased. Additionally, animal studies show that mice deficient in IL-10 or IL-4 experience 100% mortality because of enhanced Th1 polarization during the acute illness [15].

The deposited eggs can induce formation of granulomas, which are infiltrated by lymphocytes, neutrophils, macrophages and eosinophils [16]. Over time, the long-term chronic inflammation caused by eggs can lead to hepatic fibrosis [17]. However, human studies in this field are limited, and animal studies cannot fully reflect the functional and phenotypic diversity of immune cells in patients with schistosomiasis. Thus, further research is needed to explore the underlying immune mechanism. Moreover, a deeper understanding of the immune landscape in different stages of schistosome infection will not only contribute to treatment and elimination of schistosomiasis but also provide insight into the protective effects of schistosome infection in some autoimmune diseases.

Single-cell RNA sequencing has been widely used to profile the transcriptomes of immune cells in various diseases [18–21]. Although scRNA-seq has been performed to describe a single-cell atlas of schistosomes in different life stages, scRNA-seq is rarely applied to map the immune landscape in patients with schistosomiasis [22, 23]. Our study aimed to profile the immune characteristics of chronic schistosomiasis and advanced schistosomiasis by single-cell RNA sequencing, which can dissect cellular heterogeneity based on transcriptomes at the single-cell level [24]. By revealing the features of immune cells in the peripheral blood of patients with *Schistosomiasis japonica*, this study will further clarify the pathogenesis of schistosomiasis and help to identify potential targets for diagnosis and treatment of schistosome infection.

## Methods

### Human subjects

Peripheral blood mononuclear cells (PBMCs) were isolated from patients with chronic *Schistosomiasis japonica* or advanced *Schistosomiasis japonica*; PBMCs from healthy volunteers served as a control. The characteristics of the enrolled patients are shown in Additional file 1: Table S1. The diagnosis of chronic *Schistosomiasis japonica* was based on criteria including history of contaminated water exposure and praziquantel treatment, seropositivity of anti-schistosome antibodies-IgG and typical ultrasonic findings (linear strong echoes) [25]. The inclusion criteria for advanced *Schistosomiasis japonica* cases were as follows: long-term repeated history of contaminated water exposure and definitive praziquantel treatment, portal hypertension, ascites, splenomegaly or gastroesophageal variceal bleeding. The study involved several exclusion criteria, including seropositivity for hepatitis B or C virus, alcohol-induced cirrhosis, autoimmune liver or other autoimmune disease or tumor. Our study was approved by the Ethics Committee of the 3rd Xiangya Hospital of Central South University and received written informed consent.

### Single-cell RNA sequencing

Our study consisted of three groups, including patients with chronic *Schistosomiasis japonica*, advanced *Schistosomiasis japonica* and healthy controls. PBMCs were isolated by density gradient centrifugation using Ficoll-Paque Plus medium and washed with Ca/Mg-free phosphate-buffered saline (PBS). To remove the red blood cells, 2 ml GEXSCOPE^®^ red blood cell lysis buffer was added, and the sample was incubated at 25 °C for 10 min. The sample was then centrifuged at 500×*g* for 5 min, and the cells was resuspended in PBS. The samples were centrifuged at 400×*g* for 5 min at 4 °C, and the supernatant was discarded. After removing red blood cells, PBMCs were isolated by centrifugation at 400×*g* for 10 min at 4 °C. The supernatant was discarded, and the PBMCs were resuspended in PBS to obtain a single-cell suspension. Single-cell suspensions with 1 × 10^5^ cells/ml were loaded onto microfluidic devices. According to the GEXSCOPE^R^ protocol, single-cell RNA sequencing libraries were constructed using GEXSCOPE^R^ Single-Cell RNA Library Kit (Singleron Biotechnologies, Nanjing, China, Catalogue Number: 4180021), and individual libraries were pooled for sequencing after dilution to 4 nM [26]. The pooled samples were sequenced using a NovaSeq 6000 (Illumina, San Diego, CA, USA) with 150-bp paired end reads.

### scRNA-seq quantification

Raw reads were processed to generate gene expression profiles using an internal pipeline. Briefly, after filtering read one without poly T tails, the cell barcode and UMI were extracted. Adapters and poly A tails were trimmed (fastp V1) before aligning read two to GRCh38 with ensemble version 92 gene annotation (fastp 2.5.3a and featureCounts 1.6.2) [27]. Reads with the same cell barcode, UMI and gene were grouped together to calculate the number of UMIs per gene per cell. The RNA sequencing data were analyzed such as for cell type identification and clustering analysis, with the Seurat program (http://satijalab.org/seurat/, R package, v.3.2.1) [28, 29].Unique molecular identifier (UMI) count tables were loaded into R (R version 4.0.2) using the read.table function. KEGG functional enrichment analysis was performed on differentially expressed genes (DEGs) to reveal pathways significantly associated with the genes specifically expressed [30]. We used CellChat to perform cell-cell interaction analysis, which is based on known interactions among signaling ligands, receptors and their cofactors. The average gene expression of each cell type was used as input data for GSVA pathway enrichment analysis. The cell differentiation trajectory was reconstructed using Monocle2, and differentially expressed genes were used to sort cells in order of spatial-temporal differentiation.

### Flow cytometry

After peripheral blood was collected, fresh anticoagulated whole blood was stained with BD Multitest 6-color TBNK reagent (CD45-PerCP-Cy5.5, CD3-FITC, CD4-PE-Cy7, CD8-APC-Cy7, CD19-APC, CD16-PE, CD56-PE, Catalogue Number: 662967). Flow cytometry was performed using a BD FACS Canto II, and the results were analyzed using FlowJo 10.4 software (Tree Star, Ashland, OR, USA).

### Statistical analysis

The significance level was assessed by an unpaired t-test, and all data are expressed as the means ± SD. *P* values < 0.05 were considered statistically significant. Calculations were performed using GraphPad Prism software package 8.0 (GraphPad Prism, San Diego, CA, USA).

## Results

### Single-cell RNA sequencing and cell types at different stages of *Schistosomiasis japonica*

**Fig. 1:**
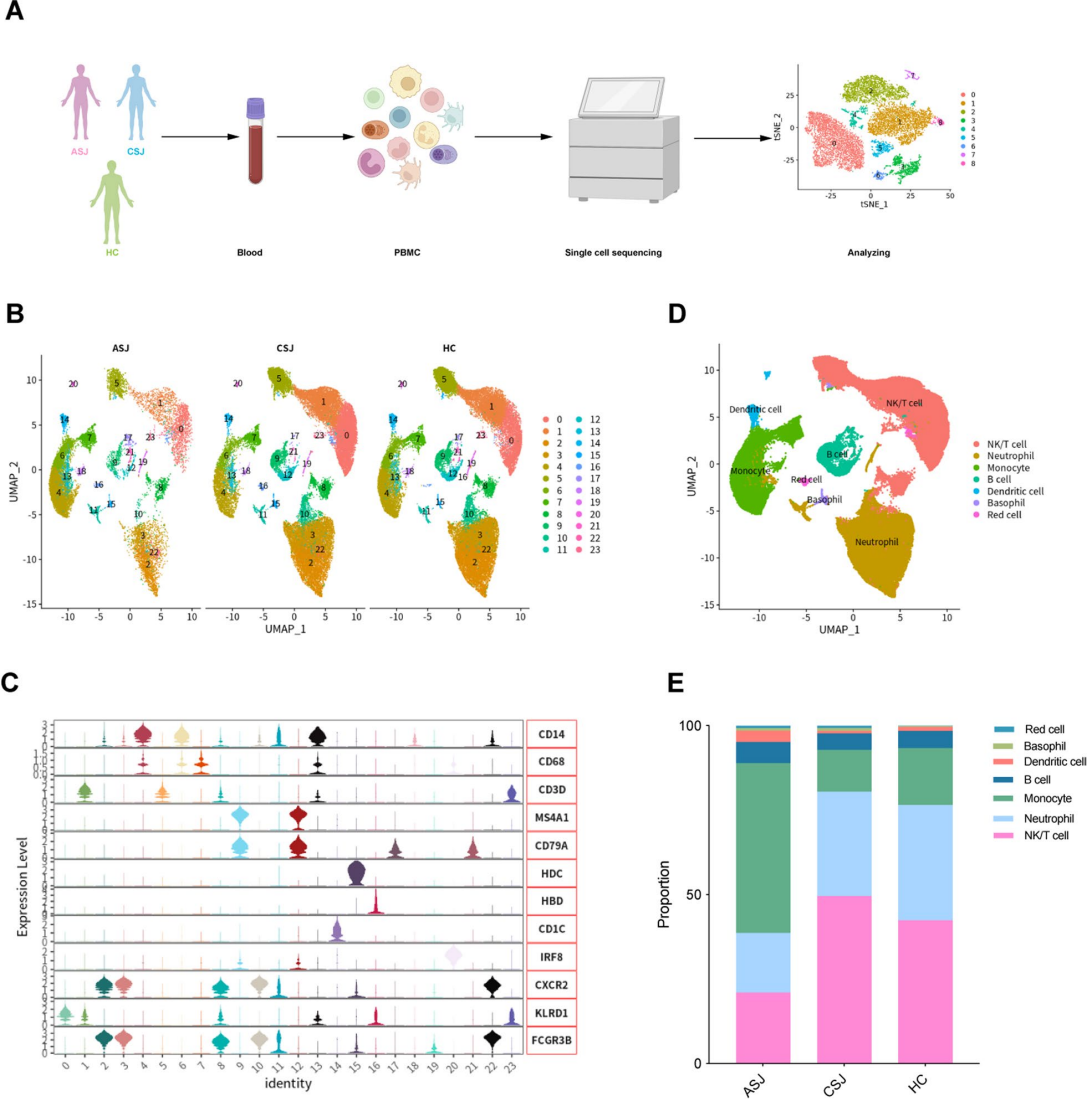
Overview of the 89,896 single cells isolated from PBMCs from three groups. (**a**) Flowchart of our study including grouping, PBMCs, sequencing and analyzing. (**b**) UMAP plot of different clusters and three groups [chronic *Schistosomiasis japonica* patients (CSJ, *n* = 4), advanced *Schistosomiasis japonica* patients (ASJ, *n* = 2) and healthy controls (HC, *n* = 4)]. (**c**) Expression of marker genes in different clusters. (**d**) UMAP plot of annotation clusters. (**e**) The percentage change tendency of each cell cluster in the three groups

PBMCs were isolated from patients with chronic *Schistosomiasis japonica* (*n* = 4; the chronic group), advanced *Schistosomiasis japonica* (*n* = 2; the advanced group) and healthy controls (*n* = 4; the HC group). Immune cells from single-cell suspensions were sequenced by single-cell RNA sequencing, and further biological analysis was performed on the sequencing data (Fig. 1a). To perform quality control (QC) analyses, cells containing < 25% mitochondrial genes were included, and cells with unique feature counts < 200 or > 10,000 were filtered out. A total of 89,896 cells were obtained for further analysis. The UMAP plot showed 24 major cell clusters in the three groups (Fig. 1b). We annotated all clusters according to marker genes and identified monocytes, NK/T cells, B cells, dendritic cells, neutrophils, basophils and red blood cells (Fig. 1c, d). By analyzing the percentage of different cells in three groups, the percentages in the advanced *Schistosomiasis japonica* group were found to be obviously different from those of the chronic *Schistosomiasis japonica* and healthy controls groups. The clusters of advanced *Schistosomiasis japonica* were composed predominantly of monocytes, while neutrophils and NK/T cells dominated in chronic *Schistosomiasis japonica* and healthy controls (Fig. 1e).

### Increased NKT cells in patients with schistosomiasis

**Fig. 2:**
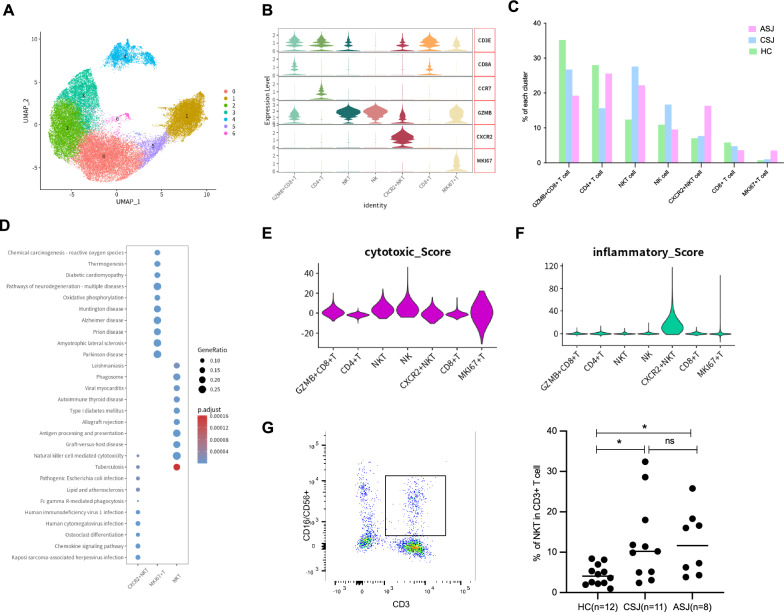
Increased NKT cells in patients with schistosomiasis. (**a**) UMAP plots of the 36,983 NK/T cells for seven clusters. (**b**) Violin plots of genes in each NK/T cells cluster. (**c**) The percentage change tendency and contribution of each NK/T cell cluster in the three groups. (**d**) KEGG pathway enrichment data of NKT cells, *CXCR2* + NKT cells and MKI67 + T cell(1). (**e, f**) Violin plots showing the cytotoxic score and inflammatory score of T cells. (**g**) The percentage of NKT cells in T cells. The significance level was assesses by an unpaired t-test, and the data are shown as the mean ± SD value (*p < 0.05; **p < 0.01; ***p < 0.001; ****p < 0.0001)

We detected 36,983 NK/T cells that were clustered into seven main clusters (Fig. 2a). Cluster (3) expressed *GZMB* but not *CD3E*, which suggested that they were NK cells. Cluster (2) and Cluster (4) were NKT cells with *GZMB* and *CD3E.* Only Cluster (2) expressed *CXCR2*, while Cluster (4) did not express it. Cytotoxic T cells (*CD3E* + *CD8* +) were classified into Cluster (0) and Cluster (5). CD8 + Cluster (5) might have weak cytotoxic activity due to the lack of *GZMB*. Cluster (6) contained T cells with strong proliferative ability and expression of *MKI67*. Cluster (1) contained CD4 + T cells (Fig. 2b). NKT cells and MIKI67 + T cells were obviously increased in the chronic group and the advanced group compared to the HC group. In particular, CXCR2 + NKT cells accounted for an important component in the advanced group (Fig. 2c). KEGG pathway analysis was performed, and the DEG-enriched pathways of CXCR2 + NKT cells are involved in natural killer cell-mediated cytotoxicity and chemokine signaling pathways; NKT cells are also involved in natural killer cell-mediated cytotoxicity and phagosomes. Oxidative phosphorylation was among the DEG-enriched pathways of MKI67 + T cells (Fig. 2d). Cytotoxic score analysis showed that NKT cells and NK cells had significant cytotoxicity, and inflammatory score analysis showed CXCR2 + NKT cells to be proinflammatory cells (Fig. 2e, f). Flow cytometry results showed significantly higher rates of NKT cells in patients with schistosomiasis (both the chronic group and the advanced group) than in the healthy controls, with no significant differences between the chronic group and the advanced group (Fig. 2g).

**Fig. 3:**
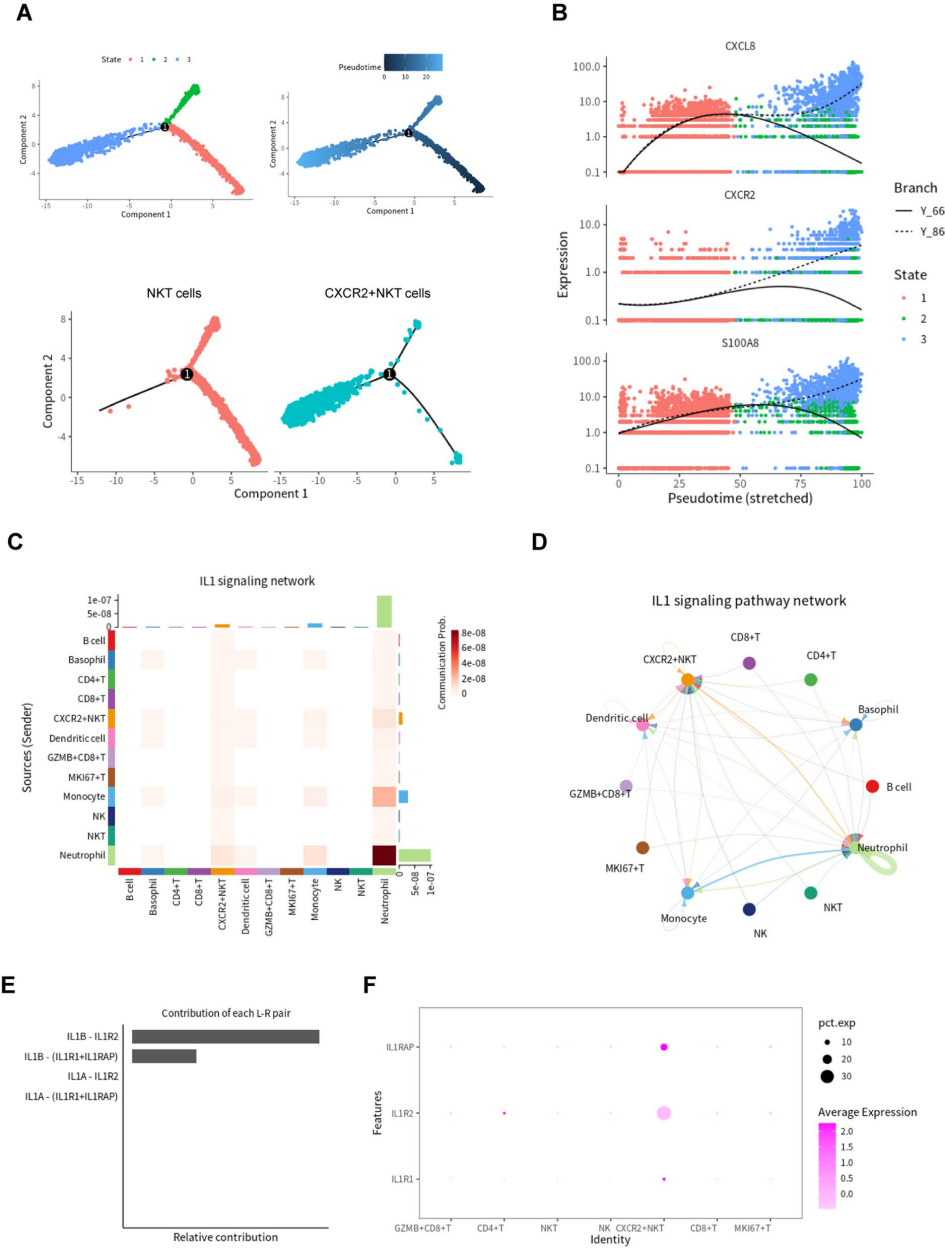
Pseudotime trajectory of T cells and CellChat between different cell clusters. (**a**) Pseudotime trajectory of NKT cells ]Cluster (2)] and CXCR2 + NKT cells [Cluster (4)]; dark blue is the start of pseudotime. (**b**) Scatter plots showing expression changes of inflammatory genes (*S100A8*, *CXCL8* and *CXCR2*) over time. (**c**) Heatmaps of the differential number of interactions between different cell clusters in the IL1 signaling network. (**d**) Circle plots displaying the IL1 signaling network between different cell clusters. (**e**) Relative contribution of each ligand-receptor pair to the IL1 signaling network. (**f**) Dot plot of *IL1R1*, *IL1R2* and *IL1RAP* in T cells

We generated a trajectory plot to explore the relationship between NKT cells [Cluster (2)] and CXCR2 + NKT cells [Cluster (4)], which included three states. Cluster ( 2) was mainly in state 1 and state 2, and Cluster (4) belonged to state 3. Pseudotime analysis showed that Cluster (4) appeared at the end of the trajectory (Fig. 3a). In the process of differentiation, proinflammatory genes (*S100A8* and *CXCL8*) were upregulated in CXCR2 + NKT cells (Fig. 3b). We utilized CellChat to analyze cell-to-cell communication between T cells and other immune cells. The results showed CXCR2 + NKT cells, neutrophils and monocytes to be closely related in the IL1 signaling pathway (Fig. 3c, d). Contribution analysis of each L − R pair showed that IL1B–IL1R2 was the most dominant L − R in the IL1 signaling pathway (Fig. 3e). The receptors of IL1B (IL1R1, IL1R2 and IL1RAP) were mainly expressed in CXCR2 + NKT cells (Fig. 3f).

### Expansion of plasma cells in advanced *Schistosomiasis japonica*

**Fig. 4:**
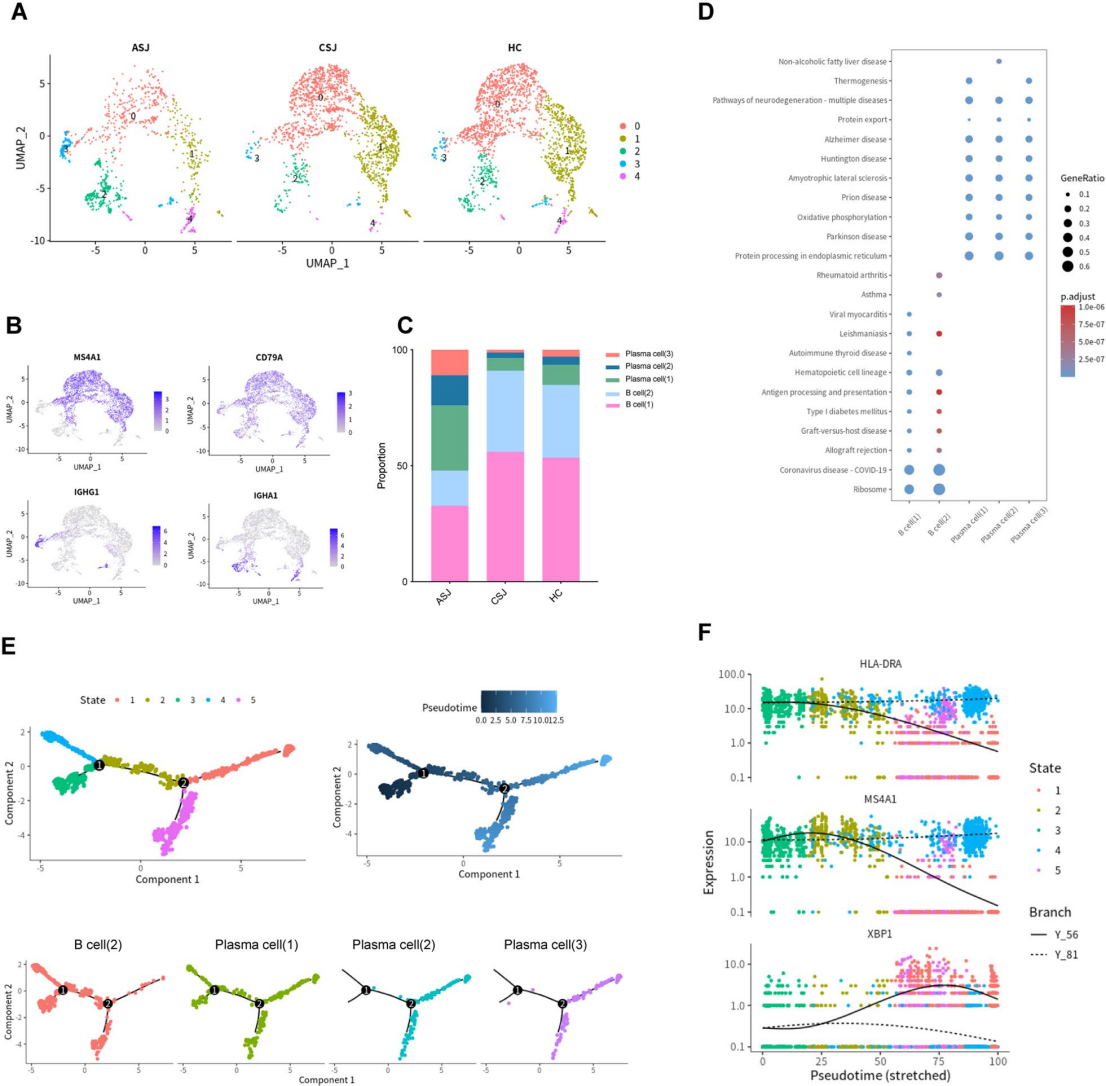
Expansion of plasma cells in advanced *Schistosomiasis japonica*. (**a**) UMAP plots of the 4717 B cells for five clusters and three groups. (**b**) Expression of marker genes of different B cells. (**c**) The percentage contribution of each B cells cluster in the three groups. (**d**) KEGG pathway enrichment data of different cells. (**e**) Pseudotime trajectory of B cells (2), plasma cells (1), plasma cells (2) and plasma cells (3); dark blue is the start of pseudotime. (**f**) Scatter plots showing the expression changes of inflammatory genes (*HLA-DRA*, *MS4A1* and *XBP1*) over time

We examined 4717 B cells, which were divided into five main clusters. The UMAP plot showed different distributions for the three groups (Fig. 4a). *CD79A* was expressed in all clusters, and *MS4A1* was mainly expressed in Cluster (0) and Cluster (1). Cluster (2), Cluster (3) and Cluster (4) contained plasma cells, characterized by high expression of *IGHA1* or *IGHG1 *(Fig. 4b). There were obviously increased plasma cells in the advanced group (Fig. 4c). The enriched KEGG pathways of B cells (1) and B cells (2) are related to ribosome and antigen processing and presentation; plasma cells (1), plasma cells (2) and plasma cells (3) are involved in protein processing in the endoplasmic reticulum, oxidative phosphorylation and protein export (Fig. 4d). We also generated a trajectory plot to investigate the relationship between B cells (2), plasma cells (1), plasma cells (2) and plasma cells (3), which included five states. Pseudotime analysis showed B cells (2) and plasma cells (1) in all states; plasma cells (2) and plasma cells (3) appeared at the two ends of trajectory branch 2 (Fig. 4e). The results showed that *HLA-DRA* and *MS4A1* were downregulated in the process of differentiation. *XBP1*, a key transcription factor for plasma cells, was found upregulated in plasma cells (Fig. 4f).

### Differential enrichment of heterogeneous monocytes in *Schistosomiasis japonica*

**Fig. 5:**
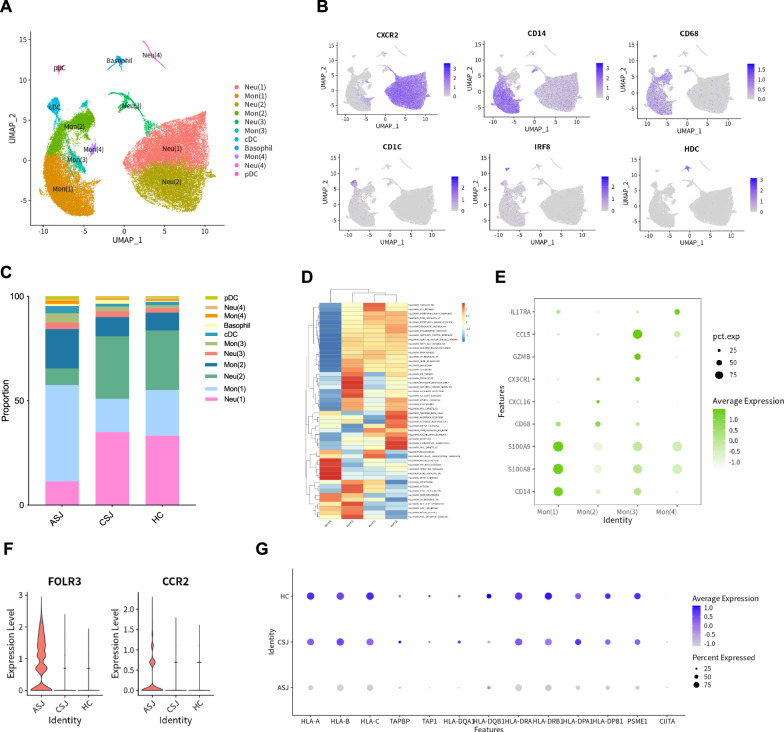
Differential enrichment of myeloid cells in *Schistosomiasis japonica*. (**a**) UMAP plots of 47,743 myeloid cells for 11 clusters. (**b**) Feature plot of marker genes of myeloid cells. (**c**) The percentage of each cluster in the three groups. (**d**) GSVA enrichment data of monocytes. (**e**) Dot plot of genes in monocytes. (**f**) Violin plots of *CCR2* and *FOLR3* in the three groups. (**g**) Bubble plots of MHC molecules in the three groups in monocytes

Myeloid cells were further investigated, and 47,743 were included in our analysis. These cells were grouped into 11 clusters. There were four clusters of neutrophils, four clusters of monocytes, one cluster of cDCs, one cluster of pDCs and one cluster of basophils (Fig. 5a, b). Monocytes were clearly increased in the advanced group (Fig. 5c). GSVA was performed to identify differences between different monocytes. Mon(1) was primarily related to the inflammatory response. Mon(2) was associated with notch signaling and TNF-α signaling via NF-κB. Mon(3) was involved in protein secretion and bile acid metabolism, and Mon(4) was related to TGF-β signaling (Fig. 5d). Inflammatory genes (*S100A8* and *S100A9*) were highly expressed in the Mon(1). *CXCL16* and *CX3CR1* were mainly shown in Mon(2). *CCL5* and *GZMB* were present in Mon(3), and *IL17RA* was highly expressed in Mon(4) (Fig. 5e). *FOLR3* and *CCR2* were predominantly expressed in monocytes in the advanced group (Fig. 5f). The advanced group showed low expression of MHC molecules (*HLA-A*, *HLA-B*, *HLA-C*, *TAPBP*, *TAP1*, *HLA-DQA1*, *HLA-DQB1*, *HLA-DRA*, *HLA-DRB1*, *HLA-DPA1*, *HLA-DPB1*, *PSME1* and *CIITA*) (Fig. 5g).

## Discussion

Schistosomiasis remains a devastating parasitic disease, especially in developing countries in sub-Saharan Africa, the Middle East, South America and Southeast Asia. With no vaccine and only one drug for treatment, there is an urgent need for further research to investigate the immune mechanisms involved in schistosomiasis and to identify potential therapeutic targets. In this study, we used scRNA-seq to explore the basic biology of immune cells in peripheral blood of patients with *Schistosomiasis japonica*. Our study for the first time to our knowledge mapped the immune cell landscape in the peripheral blood of patients with schistosomiasis, revealing the complexity and heterogeneity of immune cells in different stages of schistosome infection. This research enabled identification of stage-specific immune cells and marker genes, activation of molecular pathways and immune function evaluation. Hence, this study deepens our understanding of the immune mechanisms in schistosomiasis and provides a transcriptional atlas of peripheral immune cells facilitating elimination of schistosomiasis.

scRNA-seq is a powerful technique for providing a transcriptional atlas of immune cells and for analyzing the heterogeneity of cell populations. With its high resolution, scRNA-seq has been widely applied in various diseases [20, 31, 32]. In this study, scRNA-seq analysis revealed transcriptional signatures of various peripheral immune cells in patients in different stages of *Schistosomiasis japonica*.

Our scRNA-seq analysis identified 24 major cell clusters based on classical marker genes. Previous studies have demonstrated involvement and significance of Th1 and Th2 immune responses in mice and patients infected with *S. japonicum*; however, the cellular basis remains poorly defined [33]. Consistent with previous studies, our results revealed that different T-cell subsets are involved in the pathogenesis of different stages of *Schistosomiasis japonica*. In addition, we observed differences in other immune cells between patients with chronic schistosomiasis and advanced schistosomiasis, particularly monocytes and B cells. Although previous studies have reported their roles in schistosomiasis, the transcriptional signatures of these cells will definitely promote understanding of their roles and mechanism in *Schistosomiasis japonica* [34, 35]. Furthermore, scRNA-seq is capable of identifying rare immune cells as well as comprehensively defining the function of different immune cells. Thus, further analysis of these scRNA-seq data will contribute to identification of novel immune cells in *S. japonicum* infection.

Our analysis results showed that neutrophils accounted for the major proportion in the chronic group and the healthy group but that monocytes dominated in the advanced group. The higher level of neutrophils in chronic schistosomiasis is associated with formation of granulomas, as granulomas in patients with *Schistosomiasis japonica* have a high ratio of neutrophils [36]. However, the granulomas may become degraded in advanced schistosomiasis. Although peripheral blood-based analysis is unable to fully reveal immune cells surrounding *S. japonicum* egg-induced granulomas, scRNA-seq analysis of PBMCs contributed to our mechanistic study of the transition from chronic schistosomiasis to advanced schistosomiasis. For further investigation, we performed analyses of different cell types. NK/T cells were divided into seven main clusters. NKT cells were obviously increased in the chronic group and the advanced group compared with the HC group. There were increased CXCR2 + NKT cells in the advanced group, which had high expression of proinflammatory genes (*S100A8* and *CXCL8*). Our results showed that CXCR2 + NKT cells might migrate to the liver via chemotaxis and exhibit both cytotoxic and proinflammatory activity. Our previous study also showed that GZMB + T cells are increased in *schistosome*-associated liver fibrosis [37]. Previous studies have also demonstrated the importance of NKT cells in hepatic inflammation and fibrosis. However, the opposite effects of NKT cells occur in hepatic fibrosis in different liver diseases and stages [38–41]. On the one hand, NKT cells may inhibit activation of HSCs or kill HSCs to attenuate the proinflammatory effects and hepatic fibrosis. On the other hand, NKT cells might promote the progression of hepatic fibrosis through production of the type 2 profibrotic cytokines IL-4 and IL-13 [42–44]. In addition, NKT cells might influence the Th1/Th2 balance of the immune response in murine schistosomiasis [45]. However, our data showed low expression of *IL4*, *IL5* and *IL13* in T cells, possibly due to the difference between peripheral blood and the liver. Further investigation in NKT cells in hepatic fibrosis of human schistosomiasis is warranted.

We also analyzed myeloid cells. Monocytes were clearly increased in the advanced group. There were three different clusters of monocytes. Mon(1) had high expression of *S100A8* and *S100A9*, which might promote the inflammatory response. *CXCL16* and *CX3CR1* were mainly expressed in Mon(2), while *CCL5* and *GZMB* were mainly expressed in Mon(3). Some research has shown that macrophages can exhibit NK cell-like cytotoxic activity in a perforin/granzyme B-dependent manner [46]. *CCR2*, a promising target for treatment of liver fibrosis, was highly expressed in the advanced group and is essential for monocyte chemotaxis to the liver [47]. CCR2+ monocytes might play a profibrotic role in *schistosome*-associated liver fibrosis and also be a target for treatment of schistosomiasis. Abundant expression of *FOLR3* was observed in the advanced group. FOLR3 binds to folate and reduced folic acid derivatives and mediates delivery of 5-methyltetrahydrofolate to the interior of cells. This preliminary result offers evidence that FOLR3 might play a potential role in the development of advanced schistosomiasis, though more studies are needed to investigate this relationship. Monocytes of the advanced group, unlike those of the other two groups, showed low expression of MHC molecules (*HLA-A*, *HLA-B*, *HLA-C*, *TAPBP*, *TAP1*, *HLA-DQA1*, *HLA-DQB1*, *HLA-DRA*, *HLA-DRB1*, *HLA-DPA1*, *HLA-DPB1*, *PSME1* and *CIITA*). Monocytes are antigen-presenting cells (APCs). Our results indicated that monocytes in patients with advanced schistosomiasis are equipped with a dampened capability of antigen presentation. Although an in vitro study indicated that SEA attenuates IFN-γ-induced MHC class II expression is partly through interaction between SEA and TLR4, the mechanism of decreased MHC molecule expression in the advanced infection group remains unclear [48]. Decreased MHC molecule expression is associated with a schistosome-mediated suppression of the host immune response to evade immune attack, as MHC molecules play an important role in initiation and regulation of immune reactions [48]. Evidence has shown that patients with schistosomiasis are more prone to infection by HIV, Kaposi's sarcoma-associated herpesvirus and virulence of hepatitis B and C viruses [49]. However, the association between decreased MHC molecule expression and increased susceptibility to those viruses is not fully understood.

Our research provides a profile of the peripheral immune landscape of human *S. japonicum* infection. However, there are still several limitations in our study, including a small sample size for each group and the inability of peripheral blood to fully reflect the mechanisms of advanced schistosomiasis. Due to the small sample size of each group, our results are preliminary, and further research with larger sample sizes is needed. In conclusion, our results provide further understanding of the pathogenesis of human schistosomiasis, and the role of CCR2+ monocytes and CXCR2+ NKT cells in schistosomiasis requires further study.

### Supplementary Information

**Additional file 1.** **Table S1.** The characteristics of patients.

## Data Availability

All of data and materials are available.

## Abbreviations

*S. haematobium*: *Schistosoma haematobium*
*S. mansoni*: *Schistosoma mansoni*
*S. japonicum*: *Schistosoma japonicum*
scRNA-seq: Single-cell RNA sequencing
PBMCs: Peripheral blood mononuclear cells
NKT cell: Natural killer T cell
CXCR: C-X-C chemokine receptor
CCR: C–C chemokine receptor
MHC: Major histocompatibility complex
FOLR3: Folate receptor gamma
Th1 cell: Type 1 T helper cells
IFN-γ: Interferon gamma
IL: Interleukin
TNF-α: Tumor necrosis factor alpha
SEA: Soluble egg antigen
UMI: Unique molecular identifier
DEGs: Differentially expressed genes
GSVA: Gene set variation analysis
