# Supplementary material for: Single-cell RNA sequencing reveals a peripheral landscape of immune cells in Schistosomiasis japonica
Source: Parasit Vectors. 2023 Oct 10;16:356. doi: 10.1186/s13071-023-05975-y (PMC10563327; doi:10.1186/s13071-023-05975-y)
Supplement: Supplementary file 1 — Additional file 1. Table S1. The characteristics of patients. [file 13071_2023_5975_MOESM1_ESM.docx]

Supplementary table 1 The characteristics of patients

| Sample | Gender | Age(year) | ALT(U/L) | AST(U/L) | ALB(g/L) | TB(µmol/L) | DB(µmol/L) | Anti-SJ IgG | Ascites | | HBsAg | |
| --- | --- | --- | --- | --- | --- | --- | --- | --- | --- | --- | --- | --- |
| CSJ(1) | male | 62 | 16 | 31 | 30.6 | 35 | 16.9 | positive | negative | negative | |  |
| CSJ(2) | male | 66 | 24 | 30 | 48.1 | 14.7 | 12.2 | positive | negative | negative | |  |
| CSJ(3) | male | 54 | 40 | 39 | 40.3 | 27.4 | 13.9 | positive | negative | negative | |  |
| CSJ(4) | male | 50 | 40 | 39 | 40.3 | 27.4 | 13.9 | positive | negative | negative | |  |
| ASJ(1) | male | 53 | 8 | 25 | 42.0 | 29.5 | 6.1 | negative | positive | negative | |  |
| ASJ(2) | male | 60 | 22 | 37 | 29.1 | 18.0 | 14.4 | negative | positive | negative | |  |
| HC1 | male | 62 | 24 | 22 | 40.5 | 10.1 | 3.1 | negative | negative | negative | |  |
| HC2 | male | 65 | 27 | 27 | 46.3 | 8.1 | 2.4 | negative | negative | negative | |  |
| HC1 | female | 57 | 24 | 23 | 47.5 | 11 | 2.1 | negative | negative | negative | |  |
| HC2 | female | 54 | 14 | 22 | 46.6 | 7.5 | 2.1 | negative | negative | negative | |  |

† ALT, alanine aminotransferase; AST, aspartate aminotransferase; ALB, albumin; TB, total bilirubin; DB, direct bilirubin; SJ, Schistosomiasis Japonica.
